# Utility of comprehensive genomic profiling in directing treatment and improving patient outcomes in advanced non-small cell lung cancer

**DOI:** 10.1186/s12916-021-02089-z

**Published:** 2021-10-01

**Authors:** Shen Zhao, Zhonghan Zhang, Jianhua Zhan, Xin Zhao, Xinru Chen, Liyun Xiao, Kui Wu, Yuxiang Ma, Mengzhen Li, Yunpeng Yang, Wenfeng Fang, Hongyun Zhao, Li Zhang

**Affiliations:** 1 Department of Medical Oncology, Sun Yat-sen University Cancer Center, 651 Dongfeng East Road, Guangzhou, 510060 China; 2 State Key Laboratory of Oncology in South China, Guangzhou, China; 3 Collaborative Innovation Center for Cancer Medicine, Guangzhou, China; 4 Department of Experimental Research, Sun Yat-sen University Cancer Center, Guangzhou, China; 5 BGI-Shenzhen, Shenzhen, China; 6 Guangdong Provincial Key Laboratory of Human Disease Genomics, Shenzhen Key Laboratory of Genomics, BGI-Shenzhen, Shenzhen, China; 7 Department of Clinical Research, Sun Yat-sen University Cancer Center, 651 Dongfeng East Road, Guangzhou, 510060 China; 8 MyGene Diagnostics Co., Ltd., Guangzhou, China

**Keywords:** Precision oncology, Comprehensive genomic profiling, Genotype-matched therapies, Biomarker-selected trials, Non-small cell lung cancer

## Abstract

**Background:**

With the identification of new targetable drivers and the recent emergence of novel targeted drugs, using comprehensive genomic profiling in lieu of the routine testing for classic drivers in the clinical care for advanced NSCLC has been increasingly advocated. However, the key assumption justifying this practice, that comprehensive genomic profiling could lead to effective anticancer therapies and improve patient outcomes, remains unproved.

**Methods:**

Comprehensive genomic profiling was prospectively applied in 1564 advanced NSCLC patients to identify potentially actionable genomic alterations. Patients were assigned to genotype-matched targeted therapies or nonmatched therapies based on the profiling results. Its utility in directing treatments was determined by the proportion of patients receiving genotype-matched targeted therapies and the proportion of patients being enrolled into genotype-matched clinical trials. Its impacts on patient outcomes were assessed by comparing progression-free survival (PFS) and overall survival (OS) between patients who received a genotype-matched and nonmatched therapy.

**Results:**

From October 2016 to October 2019, tumor genomic profiles were established in 1166 patients, leading to a matched targeted therapy in 37.7% (*n* = 440) and a genotype-matched trial enrollment in 20.9% of patients (*n* = 244). Potentially actionable alterations were detected in 781 patients (67.0%). For these patients, a genomic profiling-directed matched therapy significantly improved PFS (9.0 months vs 4.9 months, *P* < 0.001) and OS (3.9 years vs 2.5 years, *P* < 0.001) compared with a nonmatched therapy. Excluding patients with standard targeted therapies, genomic profiling led to a matched targeted therapy in 16.7% (*n* = 24) and a matched trial enrollment in 11.2% (*n* = 16) of patients. No PFS (4.7 months vs 4.6 months, *P* = 0.530) or OS (1.9 years vs 2.4 years, *P* = 0.238) benefit was observed with the use of genotype-matched targeted therapies in this population.

**Conclusions:**

Comprehensive genomic profiling is of clinical utility in assisting treatment selection, facilitating clinical trial enrollment, and improving patient outcomes in advanced NSCLC. However, for patients carrying alterations without standard-of-care targeted drugs, the interpretation of genomic profiling results should be careful given the low likelihood of benefit from the investigational or off-label use of targeted therapies in this population in the current treatment landscape.

**Trial registration:**

ChiCTR1900027582 (retrospectively registered on 19 November 2019)

**Supplementary Information:**

The online version contains supplementary material available at 10.1186/s12916-021-02089-z.

## Background

Genetic testing for EGFR, ALK, and ROS1 is a standard of care for patients with advanced non-small cell lung cancer (NSCLC) [1, 2]. Recently, with the identification of new targetable drivers and the emergence of effective targeted therapies, broadly applying comprehensive genomic profiling in the clinical care for advanced NSCLC in lieu of the routine testing for classic drivers has been advocated [1, 3–5]. However, the key assumption justifying this practice, that the detection of potentially actionable alterations by comprehensive genomic profiling could lead to effective antitumor therapies and eventually improve patient outcomes, has not been proved yet [6–8].

The SHIVA study, a randomized trial comparing genomic profiling-directed targeted therapies versus conventional therapies reported a negative progression-free survival result [9]. A retrospective study also observed a lack of association between board-based genomic sequencing and survival extension in patients with advanced NSCLC [10]. Nevertheless, these studies were both conducted before 2016. Effective targeted therapies against multiple alterations including RET rearrangements, BRAF V600E, and MET exon 14 alterations have been developed since then [11–13]. Patients carrying alterations other than EGFR, ALK, and ROS1 now have increased access to targeted drugs off-label or through a clinical protocol [14]. Therefore, a re-evaluation on the clinical implications of comprehensive genomic profiling in the current treatment landscape of advanced NSCLC is warranted.

In 2016, China launched its Precision Medicine Project, intended to tackle multiple life-threatening diseases by harnessing huge amount of data from genome sequences to health records. As the National Center for Clinical Trials and Research of Anticancer Drugs, Sun Yat-sen University Cancer Center undertook part of the project and initiated a Personalized Therapy for Advanced Lung Cancer Program (PREVAIL). Here, we report our experience with prospectively applying comprehensive genomic profiling in 1564 patients to evaluate the utility of this practice in treatment selection, trial enrollment, and clinical outcome improvement for Chinese patients with advanced NSCLC.

## Methods

### Study design

Patients with treatment-naïve or previously treated advanced NSCLC, an ECOG performance status of 0–2, and adequate tumor tissue (archival FFPE or fresh biopsy) for genomic sequencing were eligible. Patients enrolled were genomically profiled to identify potentially actionable alterations and were assigned to clinical trials testing a matched targeted therapy (Additional file 1: Tab S1). Patients who carried potentially actionable alterations but were ineligible or unwilling to participate in genotype-matched trials will be treated with a genotype-matched targeted therapy or a nonmatched therapy off trial and stayed in the study for outcome analysis.

Genomic profiling was conducted in two CLIA-certified labs (MyGene, BGI-Shenzhen) using hybridization capture-based next-generation sequencing panels. Genomic alterations assessed included single nucleotide variations, insertions and deletions, copy number variations, and gene rearrangements in selected genes. The MyGene panels covered at least 22 lung cancer-related genes and the BGI panels covered 206 or 508 lung cancer-related genes (Additional file 2: Tab S2). The minimum coverage across all samples was ≥1000×. Actionability of genomic alterations and the level of evidence were determined based on the OncoKB dataset and drug approval status in mainland China [15]. Treatment allocation was based on the profiling results and inclusion criteria of associated trials. For patients carrying two or more actionable alterations, the decision was made according to the alteration with the highest actionability level. The study was conducted under an Institutional Review Board-approved protocol (protocol no. GZJZ-SB2016-010) and in accordance with the Declaration of Helsinki (ChiCTR1900027582).

### Statistical analysis

The clinical utility of comprehensive genomic profiling in treatment selection was measured by the proportion of patients receiving a genomic profiling-directed, matched targeted therapy and the proportion of patients being enrolled into a biomarker-selected clinical trial directed by their profiling results. Its impacts on patient outcomes were assessed by comparing progression-free survival (PFS) and overall survival (OS) between patients who received a matched targeted therapy and a nonmatched therapy. Stratified analyses in patients carrying alterations with different actionability levels and patients with different histologies and different timing of genomic profiling were performed to evaluate the extent of clinical benefits offered by comprehensive genomic profiling.

Patient characteristics were analyzed using the chi-squared test or Fisher’s exact test. PFS was defined as the time from the initiation of treatment to the date of progressive disease or death. OS was defined as the time from the diagnosis of advanced disease to the date of death or last follow-up. Data was updated as of 31 December 2020. Median PFS and OS estimates were generated using the Kaplan-Meier method. The log-rank test and Cox proportional hazards model were applied to evaluate the effect of covariates. *P* values were deemed statistically significant at two-sided *P* < 0.05. R version 3.5.1 was used for statistical analysis.

## Results

### Patient characteristics

**Fig. 1:**
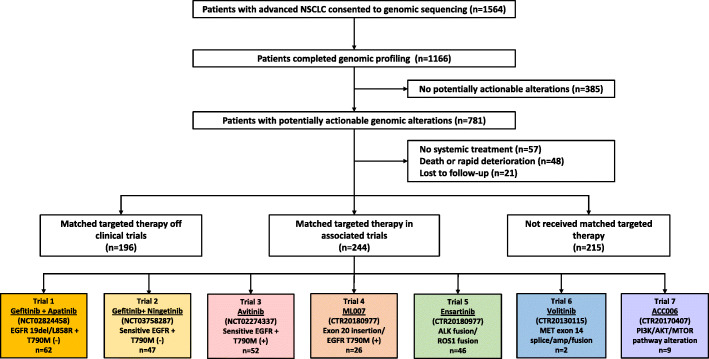
Study flow. 1L, first-line; 2L, second-line

From October 2016 to October 2019, comprehensive genomic profiling was applied to a total of 1564 patients (2094 samples). Tumor genomic profiles were established in 1968 samples from 1166 patients (74.6%, Fig. 1). The main reason for an unsuccessful genomic profiling was insufficient tumor content in the samples received. The median turnaround time from receiving samples to reporting profiling results was 5 days for samples tested in MyGene and 11 days for samples tested in BGI (Additional file 2: Tab S2). Among the patients being successfully profiled, 40% of them were females, 95% had a performance status of 0 or 1, and 59% were never smokers (Table 1). Most patients were treatment-naïve at the time of genomic profiling.

**Table 1:** Patient characteristics

No. (%)	Patients completed genomic profiling (***n*** = 1166)	Patients with potentially actionable alterations (***n*** = 781)	***P*** value
**Median age (range)**	59 (18–92)	57 (18–92)	0.034
**Sex**			0.010
Male	696 (60)	420 (54)	
Female	470 (40)	361 (46)	
**ECOG PS**			0.613
0	304 (26)	207 (27)	
1	799 (69)	524 (67)	
2	63 (5)	50 (6)	
**Histology**			< 0.001
LUAD	901 (77)	681 (87)	
LUSC	108 (9)	46 (6)	
Others^a^	157 (13)	54 (7)	
**Smoking status**			0.002
Never	684 (59)	495 (63)	
Former/current	391 (34)	207 (27)	
Unknown	91 (8)	79 (10)	
**Disease stage**			< 0.001
III	107 (9)	32 (4)	
IV	1059 (91)	749 (96)	
**Number of prior therapies**			< 0.001
Median (range)	0 (0–12)	1 (0–8)	
0	657 (56)	230 (29)	
1	204 (17)	274 (35)	
≥2	305 (26)	277 (36)	

*Abbreviations*: *ECOG PS*, the Eastern Cooperative Oncology Group performance status; *LUAD*, lung adenocarcinoma; *LUSC*, lung squamous carcinoma

^a^Others include large cell neuroendocrine carcinoma, adenosquamous carcinoma, sarcomatoid carcinoma, mucoepidermoid carcinoma, lymphoepithelioma-like carcinoma, and low differentiated tumor

### Prevalence and classification of genomic alterations

**Fig. 2:**
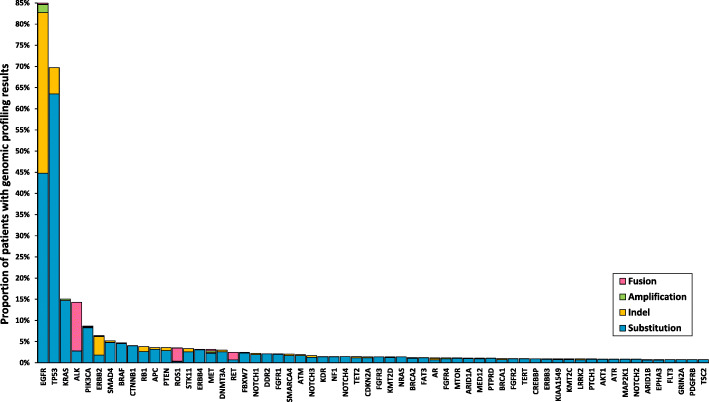
Distribution of genomic alterations among screened patients. Genomic alterations included single nucleotide variations, short and long insertions and deletions, copy number variations, and gene rearrangements. Only alterations detected in at least 10 cases are displayed

As shown in Fig. 2, alterations in EGFR were the most common in this cohort, found in 990 out of 1166 patients (84.9%), followed by alterations in TP53 (813, 69.7%), KRAS (176, 15.1%), ALK (167, 14.3%), PIK3CA (101, 8.7%), and ERBB2 (74, 6.4%). Level 1 actionable alterations defined by the OncoKB classification and drug approval status in China were sensitizing EGFR mutations, EGFR T790M mutations, ALK fusions, and ROS1 fusions. Sensitizing EGFR mutations (19del, L858R, L861Q, G719X, S768I) and EGFR T790M mutations (T790M, 19del+T790M, L858R+T790M) were found in 480 patients (41.2%). ALK fusions (EML4-ALK, WDR43-ALK, TPM3-ALK) and ROS1 fusions (CD74-ROS1, TPM3-ROS1, SDC4-ROS1) were detected in 52 (4.5%) and 23 (2.0%) patients, respectively. Level 2 actionable alterations were those who conferred tumor responses to targeted agents recently approved by the Food and Drug Administration (FDA), but not yet by the National Medical Products Administration (NMPA). These alterations included RET rearrangements, BRAF V600E, MET amplifications or exon 14 alterations, NTRK fusions, and ERBB2 mutations, which were identified in a total of 110 (9.4%) patients. Alterations ranked as level 3 or level 4 were potentially actionable targets suggested by preclinical evidence or clinical cases [15]. They were detected in 72 (6.2%) and 151 (13.0%) patients, respectively. Most level 1 and level 2 alterations were mutually exclusive, while level 3 and level 4 alterations could co-occur with other actionable targets.

Altogether, potentially actionable alterations were detected in 67% of patients (781/1166). Among them, 440 patients were tested in MyGene, 193 were tested in BGI, and 148 were tested in both labs. The alteration detection rates were similar between MyGene (588/915, 64%) and BGI (341/512, 67%, *P* = 0.374). Compared with the screening population, there was an enrichment of younger patients (*P* = 0.034), females (*P* = 0.010), lung adenocarcinomas (*P* < 0.001), and never smokers (*P* = 0.002) in patients carrying potentially actionable alterations (Table 1). Most patients were previously treated, and 36% of them had progressed after at least two prior lines of therapies.

### Treatments received

Among patients with potentially actionable alterations identified by comprehensive genomic profiling, 244 patients were enrolled into associated trials testing a matched targeted therapy, 196 received a matched targeted therapy off trial, and 215 received a nonmatched therapy (Fig. 1). Overall, comprehensive genomic profiling led to a matched targeted therapy in 37.7% (440/1166) of patients and a matched clinical trial enrollment in 20.9% (244/1166) of patients.

**Fig. 3:**
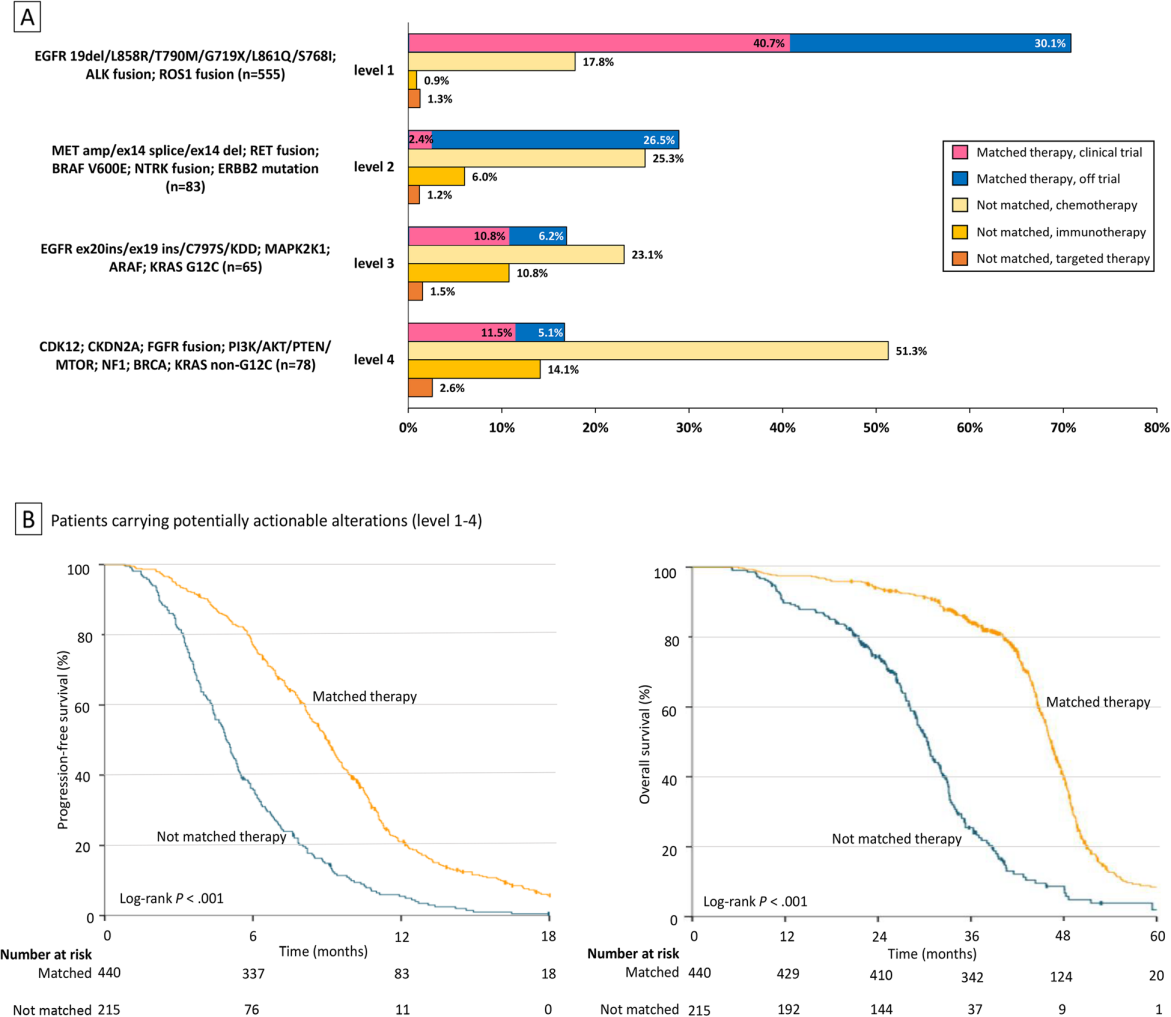
Distribution of treatments and its association with clinical outcomes measured by PFS and OS. **A** Distribution of treatments in patients carrying alterations with different actionability levels. **B** PFS and OS in patients carrying potentially actionable alterations who were treated with a matched therapy and a nonmatched therapy

Figure 3A demonstrates the treatment distribution in patients carrying alterations with different actionability levels. Patients carrying two or more actionable alterations were classified based on the alteration with the highest actionability level. For patients with level 1 actionable alterations (*n* = 555), a genomic profiling-directed targeted therapy was offered to 70.8% of patients (*n* = 393). And 40.7% of them (*n* = 226) were enrolled into an associated clinical trial testing a novel targeted agent (trial 5) or a new combination therapy (trial 1, trial 2). Ongoing chemotherapy was the most common reason that a matched targeted therapy was not used in these patients.

In patients carrying level 2–4 alterations (*n* = 226), comprehensive genomic profiling led to a matched targeted therapy in 21.2% (*n* = 48) of patients and a matched trial enrollment in 8% (18/226) of patients. Specifically, the proportion of patients receiving a genomic profiling-directed targeted therapy in patients carrying level 2, level 3, and level 4 alterations was 28.9%, 16.9%, and 16.7%, respectively. Most patients carrying level 2 alterations received a matched targeted therapy off trial (RET rearrangement: cabozantinib; BRAF V600E: dabrafenib, cabozantinib, dabrafenib + trametinib; ERBB2 mutation: afatinib, pyrotinib, dacomitinib, trastuzumab + chemotherapy). The majority of patients with level 3–4 alterations were treated with a matched targeted therapy in associated clinical trials (trial 3, trial 4, trial 7), while some were off trial (EGFR ex20ins: osimertinib 160 mg, osimertinib/afatinib + cetuximab; MTOR: everolimus). No patient carrying alterations in MAPK2K1, ARAF, KRAS, CDK12, CDKN2A, FGFR, or NF1 as their highest actionable targets received a matched targeted therapy.

In terms of nonmatched therapies, chemotherapy remained the mainstay of treatment across all actionability levels. Notably, patients carrying level 3–4 alterations were more likely to receive immunotherapies compared with patients carrying level 1–2 alterations (*P* = 0.001).

### Clinical outcomes

At the time of data cutoff (31 December 2020), the median follow-up time from the diagnosis of advanced diseases was 12.7 months (range, 1.0–111.5). Characteristics of patients with potentially actionable alterations were similar whether or not they received a matched targeted therapy (Additional file 3: Tab S3). Patients with potentially actionable alterations and a matched targeted therapy had a median PFS of 9.0 months and a median OS of 3.9 years, while patients with actionable alterations but a nonmatched therapy had a median PFS of 4.9 months and a median OS of 2.5 years (PFS: log-rank *P* < 0.001; Cox model HR = 0.38 [95% CI, 0.32–0.45], *P* < 0.001; OS: log-rank *P* < 0.001; Cox model HR = 0.24 [95% CI, 0.20–0.30], *P* < 0.001; Fig. 3B).

In multivariate analysis accounting for age, stage, ECOG PS, histology, number of prior therapies, and treatment strategies, independent factors that correlated with a better PFS were fewer lines of prior treatments (HR = 0.37 [95% CI, 0.30–0.44], *P* < 0.001) and a matched targeted therapy (HR = 0.45 [95% CI, 0.38–0.53], *P* < 0.001). In terms of OS, independent prognostic factors included better performance status (HR = 0.84 [95% CI, 0.70–0.99], *P* = 0.048) and a genotype-matched targeted therapy (HR = 0.23 [95% CI, 0.19–0.28], *P* < 0.001). With regard to the timing of genomic profiling, we noticed that matched targeted therapies directed by frontline genomic profiling demonstrated a greater impact on extending PFS (HR = 0.26 [95% CI, 0.17–0.39], *P* < 0.001) and OS (HR = 0.09 [95% CI, 0.06–0.15], *P* < 0.001) compared with the genomic profiling-directed targeted therapies in subsequent lines of treatment (PFS: HR = 0.54 [95% CI, 0.45–0.66], *P* < 0.001; OS: HR = 0.30 [95% CI, 0.23–0.37], *P* < 0.001).

**Fig. 4:**
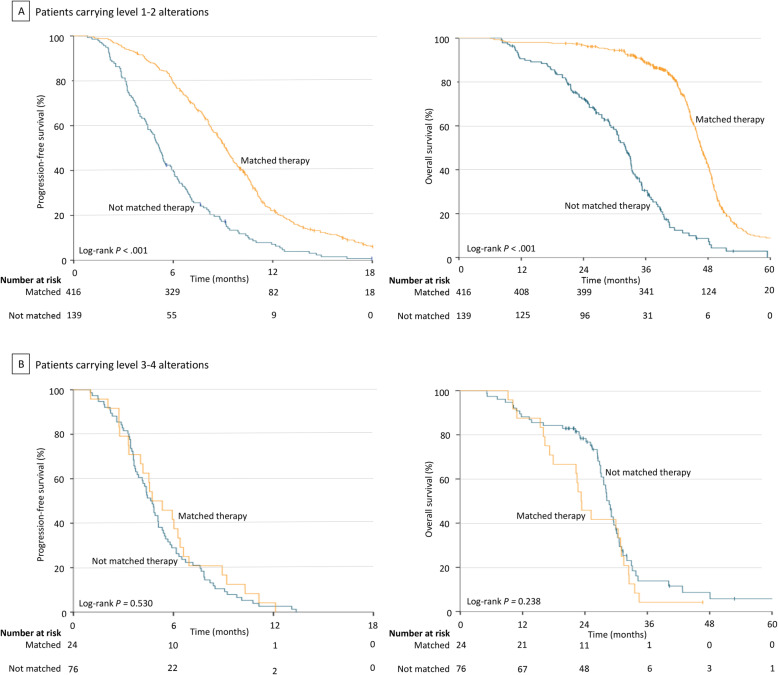
Stratified analysis in patients carrying alterations with different actionability levels. **A** PFS and OS in patients carrying level 1–2 alterations who were treated with a matched therapy and a nonmatched therapy. **B** PFS and OS in patients carrying level 3–4 alterations who were treated with a matched therapy and a nonmatched therapy

In stratified analyses based on the actionability level of alterations, a matched targeted therapy directed by comprehensive genomic profiling significantly extended PFS and OS in patients carrying level 1–2 alterations in comparison to a nonmatched therapy (PFS 9.2 months vs 5.2 months, log-rank *P* < 0.001; Cox model HR = 0.39 [95% CI, 0.32–0.48], *P* < 0.001; OS 3.9 years vs 2.7 years, log-rank *P* < 0.001; Cox model HR = 0.20 [95% CI, 0.16–0.26], *P* < 0.001; Fig. 4A). Nevertheless, no PFS or OS improvement was observed with the use of a genomic profiling-directed targeted therapy in patients carrying level 3–4 alterations compared with a nonmatched therapy (PFS 4.7 months vs 4.6 months, log-rank *P* = 0.530; Cox model HR = 0.86 [95% CI, 0.54–1.37], *P* = 0.533; OS 1.9 years vs 2.4 years, log-rank *P* = 0.238; Cox model HR = 1.35 [95% CI, 0.82–2.20], *P* = 0.240; Fig. 4B). Similar results were found in subgroup patients with different histologies (Additional file 4: Fig S1). The timing of comprehensive genomic profiling (treatment-naïve vs previously treated) did not change the results either (Additional file 5: Fig S2).

## Discussion

With the rapid development in cancer genomics and anticancer therapeutics, multiple studies have reported cases of patients who developed marked tumor responses to targeted drugs suggested by genetic testing results [6, 16]. Encouraged by these reports, comprehensive genomic profiling, a method that allows the simultaneous detection of multiple potentially actionable alterations, has been increasingly applied in routine cancer care [8, 17]. Compared with the conventional methods that test aberrations sequentially, genomic profiling with next-generation sequencing panels improved the tissue use efficiency without significantly increasing the turnaround time and cost [17–19]. Nevertheless, the clinical implication of this practice, especially its impact on patient outcomes, remains unknown in advanced NSCLC.

In this study, by prospectively applying comprehensive genomic profiling in 1564 advanced NSCLC patients, we demonstrated the utility of this practice in assisting treatment selection and facilitating enrollment of biomarker-selected clinical trials. For patients with level 1–2 actionable alterations detected by genomic profiling, a matched targeted therapy significantly extended their PFS and OS compared with a nonmatched therapy. However, for patients with level 3–4 alterations as their highest actionable targets, no marked clinical advantage was observed with the investigational or off-label use of genotype-matched targeted therapies. The clinical utility of genomic profiling in this population may still be limited by the lack of targeted drugs with satisfactory activities.

In the present cohort, potentially actionable alterations were detected by comprehensive genomic profiling in 67% of Chinese advanced NSCLC patients. This percentage was comparable with the Caucasian population, where previous studies reported a 64% and an 86.9%, respectively [20, 21]. However, the distribution of actionable alterations in Chinese patients was quite different, manifested by the higher prevalence of EGFR, ALK, and ERBB2 alterations. There were 41.2% of patients carrying sensitizing EGFR mutations or EGFR T790M mutations, which were much higher than the Caucasian patients as previously reported [20, 21]. Meanwhile, the proportion of never smokers in our cohort was also higher than the usual NSCLC patient population [22], reflecting that patients with less tobacco exposure might be more likely and more willing to use comprehensive genomic profiling.

Overall, comprehensive genomic profiling led to a matched targeted therapy in 37.7% and a matched trial enrollment in 20.9% of patients. In a stratified analysis based on actionability levels, the proportion of a genomic profiling-directed targeted therapy was 68.4% (417/638) in patients with level 1–2 alterations and 16.7% (24/143) in patients with level 3–4 alterations. The rate of being enrolled into a matched clinical trial directed by genomic profiling was 35.7% (228/638) in patients with level 1–2 alterations and 11.2% (16/143) in those with level 3–4 alterations. These data suggest the utility of comprehensive genomic profiling in increasing patient access to targeted treatments and facilitating clinical development of innovative drugs targeting less common oncogenic drivers [1]. The relatively high trial enrollment rate here may also be partly attributed to China’s recent effort to boost drug innovation, which has led to the galloping increase in the number of investigational new drugs and clinical trials. Nevertheless, we should be aware that this study was conducted in a specialized cancer center. As the National Center for Clinical Trials and Research of Anticancer Drugs, Sun Yat-Sen University Cancer Center have abundant resources on new drug research. Patients treated here tended to have a higher uptake of advanced therapeutics and clinical trials. Therefore, the proportion of patients receiving genotype-matched targeted therapies and the trial enrollment rate in this study might be higher than those in a community setting. Additionally, the vast difference between patients carrying level 1–2 and level 3–4 alterations here underlined the problem that patients with less common oncogenic drivers still had limited access to targeted treatments and few options of matched clinical trials. Studies in the USA and Japan reported similar data [18, 21, 23, 24], indicating it being a common problem that compromised the clinical utility and benefit of comprehensive genomic profiling.

Meanwhile, we noticed that tumor genomic profiling might also affect the application of immunotherapies. We previously reported that comprehensive genomic profiling could identify novel genetic predictors for tumor responses to immune checkpoint inhibitors in NSCLC [25]. In this study, patients carrying level 3–4 alterations were more likely to receive immunotherapies in comparison to those with level 1–2 alterations (*P* = 0.001). Several level 1–2 alterations had been reported to predict resistance to PD-1/PD-L1 inhibitors [26]. These findings suggested that with our improved understanding of molecular alterations and their implications, the clinical utility of comprehensive genomic profiling could go beyond targeted therapies and be further expanded in the era of immuno-oncology.

In terms of clinical outcomes, a genomic profiling-directed matched therapy significantly extended PFS and OS in patients carrying actionable alterations in comparison to a nonmatched therapy. However, for patients carrying level 3–4 alterations as their highest actionable targets, the investigational or off-label use of targeted therapies suggested by comprehensive genomic profiling failed to improve patient outcomes measured by PFS and OS. A genomic profiling-directed targeted therapy in this population even yielded a numerically shorter OS compared with a nonmatched therapy (1.9 years vs 2.4 years, *P* = 0.238). Reasons for this phenomenon may include limited access to targeted agents, low antitumor activities of existed drugs, and the efficacy of immunotherapies in these patients. Collectively, data presented here demonstrated the role of comprehensive genomic profiling in improving patient outcomes and supported its clinical application in patients with advanced NSCLC. However, for patients carrying level 3–4 actionable alterations, the benefit of genomic profiling-directed therapies may still be limited in the current treatment landscape. The interpretation of genomic profiling results in this population should be cautious given its low likelihood of clinical benefit.

The study has several limitations. Firstly, this is a nonrandomized study. Although potential confounding factors were accounted for in the statistical analysis, randomized trials are required to further validate our findings. Secondly, limited by the drug approval status and innovative drug availability in China, the latest FDA-approved drugs targeting alterations in BRAF, MET, RET, and NTRK were not available to most patients in this study. Increased availability of novel targeted drugs may further expand the utility of comprehensive tumor profiling in clinical care. Finally, NGS panels of different sizes were used in this study because the labs have developed their panels during the past years.

## Conclusions

Despite the above limitations, this study demonstrated that applying comprehensive genomic profiling in the routine care for advanced NSCLC could be justified by its utility in assisting treatment selection, facilitating trial enrollment, and improving patient outcomes. However, given the low likelihood of benefit from the investigational or off-label use of targeted therapies, the interpretation of genomic profiling results in patients carrying level 3–4 alterations should be very careful in the current treatment landscape.

## Supplementary Information

**Additional file 1: Table S1.** List of associated clinical trials in the study.

**Additional file 2: Table S2.** NGS panels and number of samples being tested.

**Additional file 3: Table S3.** Baseline characteristics between patients carrying potentially actionable alterations treated with a matched (n = 440) and nonmatched therapy (n = 215).

**Additional file 4: Figure S1.** Stratified analysis in patients with different histologies who carried alterations with different actionability levels. A. Subgroup of lung adenocarcinoma: PFS and OS in patients carrying level 1-2 alterations treated with a matched therapy and a nonmatched therapy. B. Subgroup of lung adenocarcinoma: PFS and OS in patients carrying level 3-4 alterations treated with a matched therapy and a nonmatched therapy. C. Subgroup of other NSCLC histologies: PFS and OS in patients carrying level 1-2 alterations treated with a matched therapy and a nonmatched therapy. D. Subgroup of other NSCLC histologies: PFS and OS in patients carrying level 3-4 alterations treated with a matched therapy and a nonmatched therapy.

**Additional file 5: Figure S2.** Stratified analysis in patients with different timing of genomic profiling who carried alterations with different actionability levels. A. Treatment-naïve when genomically profiled: PFS and OS in patients carrying level 1-2 alterations treated with a matched therapy and a nonmatched therapy. B. Previously treated when genomically profiled: PFS and OS in patients carrying level 1-2 alterations treated with a matched therapy and a nonmatched therapy. C. Previously treated when genomically profiled: PFS and OS in patients carrying level 3-4 alterations treated with a matched therapy and a nonmatched therapy.

**Additional file 6.** Study protocol and amendment records.

## Data Availability

Genomic sequencing data in the study are available at China National Center for Bioinformation (http://bigd.big.ac.cn/) under the project number: PRJCA003514.

## Abbreviations

ALK: Anaplastic lymphoma kinase
CLIA: Clinical laboratory improvement amendments
ECOG: The Eastern Cooperative Oncology Group
EGFR: Epidermal growth factor receptor
FDA: Food and Drug Administration
FFPE: Formalin-fixed paraffin-embedded
HR: Hazard ratio
NMPA: National Medical Products Administration
NSCLC: Non-small cell lung cancer
OS: Overall survival
PD-1/PD-L1: Programmed death 1/programmed death ligand 1
PFS: Progression-free survival
PS: Performance status
ROS1: c-ROS oncogene 1 receptor kinase
