# Supplementary material for: Utility of comprehensive genomic profiling in directing treatment and improving patient outcomes in advanced non-small cell lung cancer
Source: BMC Med. 2021 Oct 1;19:223. doi: 10.1186/s12916-021-02089-z (PMC8485523; doi:10.1186/s12916-021-02089-z)
Supplement: Supplementary file 1 — Additional file 1: Table S1. List of associated clinical trials in the study. [file 12916_2021_2089_MOESM1_ESM.docx]

**Additional file 1: Table S1. List of associated clinical trials in the study**

| **Arm** | **Trial Identifier** | **Investigated Agents** | **Trial Name** |
| --- | --- | --- | --- |
| 1 | NCT02824458 | Gefitinib+Apatinib | A Study of gefitinib with or without apatinib in patients with advanced non-squamous non-small cell lung cancer harboring EGFR mutations |
| 2 | NCT03758287 | Gefitinib+Ningetinib | Ningetinib (CT053PTSA) plus gefitinib in stage IIIB or IV NSCLC patients with EGFR mutation and T790M Negative |
| 3 | NCT02274337 | Avitinib | Safety, tolerability, pharmacokinetics and anti-tumour activity of AC0010 in advanced non-small cell lung cancer |
| 4 | CTR20180977 | ML-007 | Safety, tolerability and pharmacokinetics of ML-007 in advanced non-small cell lung cancer |
| 5 | NCT02959619 | Ensartinib (X-396) | X-396 in patients with advanced ALK-positive NSCLC: a phase 1, open-label, dose-escalating and cohort expansion study |
| 6 | CTR20130115 | Volitinib (HMPL-504) | Safety, tolerability and pharmacokinetics of volitinib (HMPL-504) in patients with advanced solid tumors |
| 7 | CTR20170407 | ACC006 | Safety, tolerability and pharmacokinetics of ACC006 in advanced solid tumors |

**Source:** [www.clinicaltrials.gov](http://www.clinicaltrials.gov); [www.chinadrugtrials.org.cn](http://www.chinadrugtrials.org.cn/index.html).
