# Supplementary material for: Utility of comprehensive genomic profiling in directing treatment and improving patient outcomes in advanced non-small cell lung cancer
Source: BMC Med. 2021 Oct 1;19:223. doi: 10.1186/s12916-021-02089-z (PMC8485523; doi:10.1186/s12916-021-02089-z)
Supplement: Supplementary file 2 — Additional file 2: Table S2. NGS panels and number of samples being tested. [file 12916_2021_2089_MOESM2_ESM.docx]

**Additional file 2: Table S2**

1. **NGS panels and number of samples being tested**
2. **Sequencing platforms and coverage**

| **Lab** | **NGS panel size** | **Number of samples tested** |
| --- | --- | --- |
| MyGene | 22 | 376 |
| MyGene | 24 | 717 |
| MyGene | 25 | 3 |
| MyGene | 52 | 149 |
| MyGene | 143 | 65 |
| BGI | 206 | 359 |
| BGI | 508 | 299 |

| **Lab** | **NGS platforms** | **Median coverage** |
| --- | --- | --- |
| MyGene | Ion PGM | 1000 |
| MyGene | Ion S5 | 1000 |
| BGI | BGI-500 | 2677.27 |
| BGI | HiSeq-5000 | 2657.58 |
| BGI | MGISEQ-2000 | 3601.18 |
