# Supplementary material for: Utility of comprehensive genomic profiling in directing treatment and improving patient outcomes in advanced non-small cell lung cancer
Source: BMC Med. 2021 Oct 1;19:223. doi: 10.1186/s12916-021-02089-z (PMC8485523; doi:10.1186/s12916-021-02089-z)
Supplement: Supplementary file 3 — Additional file 3: Table S3. Baseline characteristics between patients carrying potentially actionable alterations treated with a matched (n = 440) and nonmatched therapy (n = 215). [file 12916_2021_2089_MOESM3_ESM.docx]

**Additional file 3: Table S3.** **Baseline characteristics between patients carrying potentially actionable alterations treated with a matched and nonmatched therapy.**

| **No (%)** | **Matched therapy**  **(n=440)** | **No matched therapy (n=215)** | ***P* value** |
| --- | --- | --- | --- |
| **Median age (range)** | 55 (19-79) | 56 (18-92) | 0.635 |
| **Sex** | | | 0.196 |
| Male | 224 (51) | 121 (56) |  |
| Female | 216 (49) | 94 (44) |  |
| **ECOG PS** | | | 0.659 |
| 0 | 110 (25) | 53 (25) |  |
| 1 | 295 (67) | 149 (69) |  |
| 2 | 35 (8) | 13 (6) |  |
| **Histology** |  |  | 0.067 |
| LUAD | 329 (89) | 190 (88) |  |
| LUSC | 23 (5) | 19 (9) |  |
| Others* | 25 (6) | 6 (3) |  |
| **Smoking status** | | | 0.085 |
| Never | 327 (74) | 146 (68) |  |
| Former/Current | 113 (26) | 69 (32) |  |
| **Disease stage** | | | 0.513 |
| III | 19 (4) | 7 (3) |  |
| IV | 421 (96) | 208 (97) |  |
| **Number of prior therapies** | | | 0.425 |
| Median (range) | 1 (0-8) | 1 (0-7) |  |
| 0 | 187 (43) | 80 (37) |  |
| 1 | 138 (31) | 75 (35) |  |
| ≥2 | 115 (26) | 60 (28) |  |

*Others include large cell neuroendocrine carcinoma, adenosquamous carcinoma, sarcomatoid carcinoma, mucoepidermoid carcinoma, lymphoepithelioma-like carcinoma and low differentiated tumor.

Abbreviation: ECOG PS, the Eastern Cooperative Oncology Group performance status; LUAD, lung adenocarcinoma; LUSC, lung squamous carcinoma.
