# Supplementary material for: Utility of comprehensive genomic profiling in directing treatment and improving patient outcomes in advanced non-small cell lung cancer
Source: BMC Med. 2021 Oct 1;19:223. doi: 10.1186/s12916-021-02089-z (PMC8485523; doi:10.1186/s12916-021-02089-z)

Supplementary Figure 1

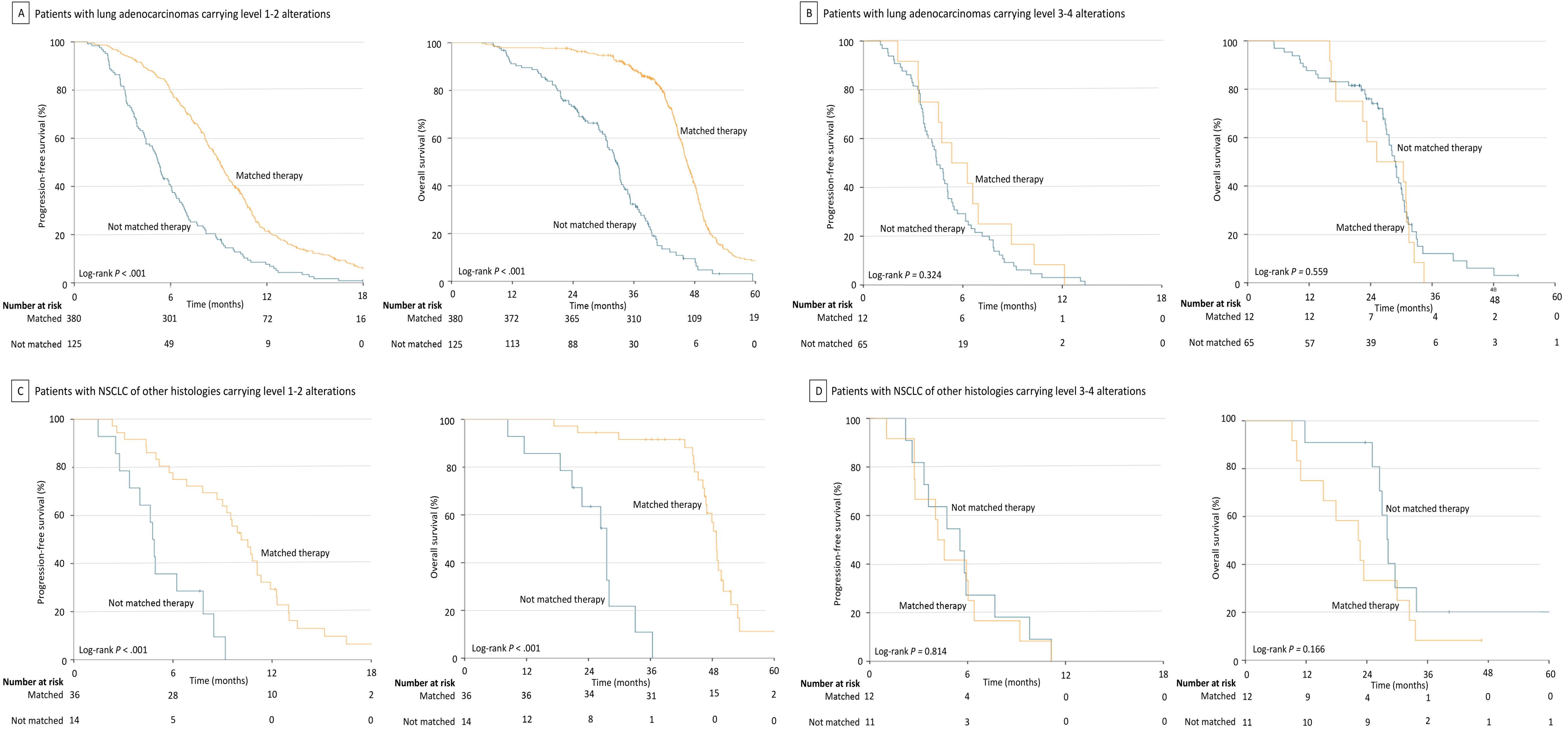

Supplement: Supplementary file 4 — Additional file 4: Figure S1. Stratified analysis in patients with different histologies who carried alterations with different actionability levels. A. Subgroup of lung adenocarcinoma: PFS and OS in patients carrying level 1-2 alterations treated with a matched therapy and a nonmatched therapy. B. Subgroup of lung adenocarcinoma: PFS and OS in patients carrying level 3-4 alterations treated with a matched therapy and a nonmatched therapy. C. Subgroup of other NSCLC histologies: PFS and OS in patients carrying level 1-2 alterations treated with a matched therapy and a nonmatched therapy. D. Subgroup of other NSCLC histologies: PFS and OS in patients carrying level 3-4 alterations treated with a matched therapy and a nonmatched therapy. [file 12916_2021_2089_MOESM4_ESM.pdf]
