# Supplementary material for: Utility of comprehensive genomic profiling in directing treatment and improving patient outcomes in advanced non-small cell lung cancer
Source: BMC Med. 2021 Oct 1;19:223. doi: 10.1186/s12916-021-02089-z (PMC8485523; doi:10.1186/s12916-021-02089-z)

Supplementary Figure 2

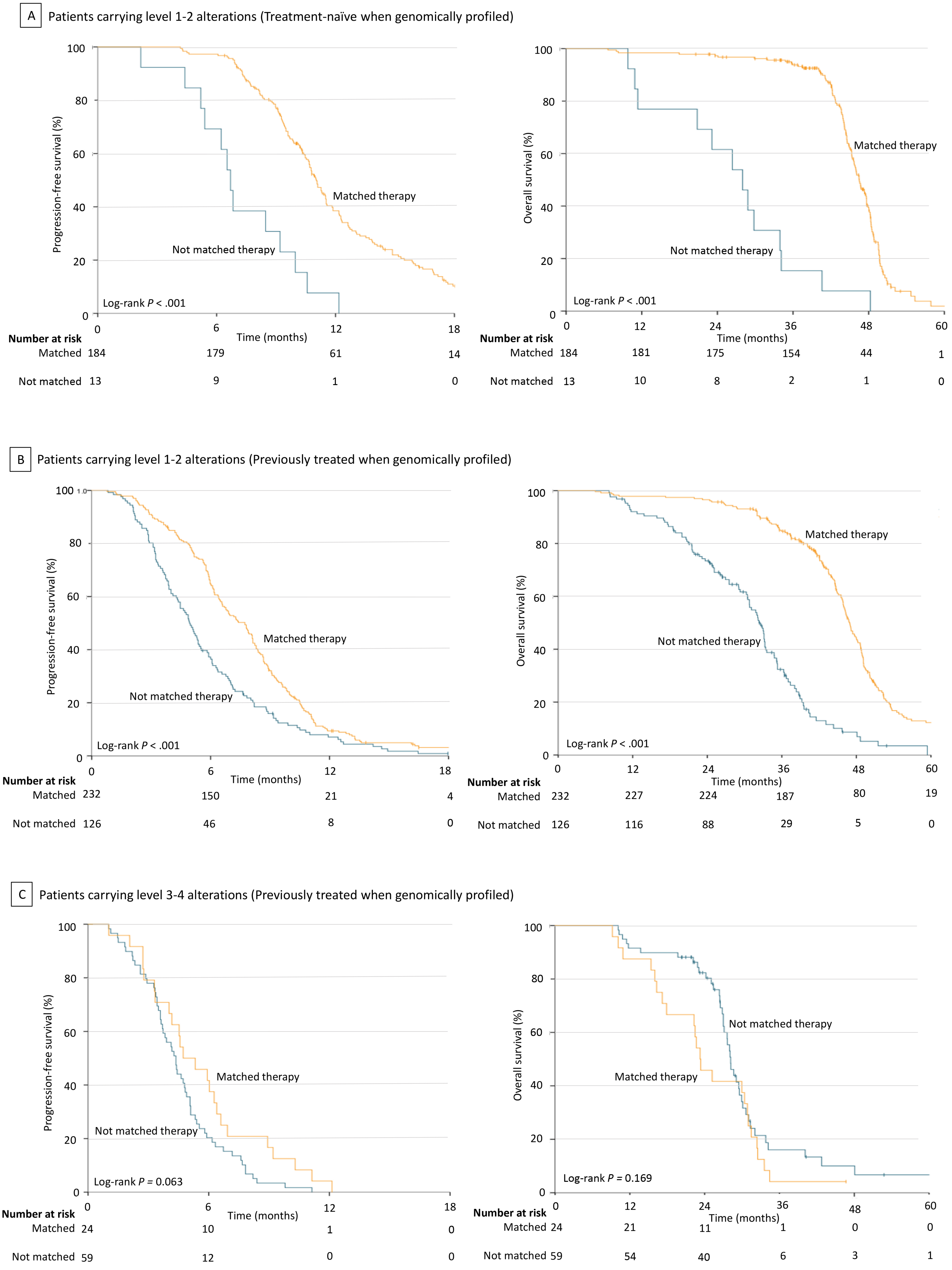

Supplement: Supplementary file 5 — Additional file 5: Figure S2. Stratified analysis in patients with different timing of genomic profiling who carried alterations with different actionability levels. A. Treatment-naïve when genomically profiled: PFS and OS in patients carrying level 1-2 alterations treated with a matched therapy and a nonmatched therapy. B. Previously treated when genomically profiled: PFS and OS in patients carrying level 1-2 alterations treated with a matched therapy and a nonmatched therapy. C. Previously treated when genomically profiled: PFS and OS in patients carrying level 3-4 alterations treated with a matched therapy and a nonmatched therapy. [file 12916_2021_2089_MOESM5_ESM.pdf]
