# Supplementary material for: Utility of comprehensive genomic profiling in directing treatment and improving patient outcomes in advanced non-small cell lung cancer
Source: BMC Med. 2021 Oct 1;19:223. doi: 10.1186/s12916-021-02089-z (PMC8485523; doi:10.1186/s12916-021-02089-z)
Supplement: Supplementary file 6 — Additional file 6. Study protocol and amendment records. [file 12916_2021_2089_MOESM6_ESM.docx]

**PREVAIL Program: Personalized Therapy for Patients with Advanced Lung Cancer**

**Study Protocol V1.0**

**GZJZ-SB2016-010**

**Principal Investigator: Li Zhang**

**Professor of Medical Oncology, Director, Department of Medical Oncology, Sun Yat-Sen University Cancer Center**

**651 Dongfeng East Road, Guangzhou, China, 510060**

**Tel: 86-20-87343458**

**E-mail:** [**zhangli@sysucc.org.cn**](mailto:zhangli@sysucc.org.cn)

**LEAD INVESTIGATORS AND SITES**

| **Arm** | **Trial Identifier** | **Investigated Agents** | **Lead Investigators** | **Lead Site** |
| --- | --- | --- | --- | --- |
| 1 | NCT02824458 | Gefitinib+Apatinib | Hongyun Zhao | Sun Yat-Sen University Cancer Center |
| 2 | NCT03758287 | Gefitinib+Ningetinib | Li Zhang | Sun Yat-Sen University Cancer Center |
| 3 | NCT02274337 | Avitinib | Li Zhang | Sun Yat-Sen University Cancer Center |
| 4 | CTR20180977 | ML007 | Li Zhang | Sun Yat-Sen University Cancer Center |
| 5 | NCT02959619 | Ensartinib | Li Zhang | Sun Yat-Sen University Cancer Center |
| 6 | CTR20130115 | Volitinib | Lin Shen | Beijing Cancer Hospital |
| 7 | CTR20170407 | ACC006 | Wenfeng Fang  Su Li | Sun Yat-Sen University Cancer Center,  Sun Yat-Sen University Cancer Center |

**LABORATORY SITES AND CONTACTS**

**MyGene Diagnostics Co., Ltd.**

Contact: Mengzhen Li, PhD

C101, 9 Spiral 4^th^ Road, Guangzhou International Biological Island, 510320, Guangzhou, China

Tel: 86-20-89637615

E-mail: limengzhen@maijinggene.com

**Shenzhen Key Laboratory of Genomics, BGI-Shenzhen**

Contact: Xin Zhao, PhD

BGI-Shenzhen, Shenzhen 518083, China

Tel: 86-0755-36307888

E-mail: zhaoxin@genomics.cn

**PREVAIL Program**

**Protocol Amendments Records**

| **Version number** | **Version date** | **Amendment part and reasons** |
| --- | --- | --- |
| 1.0 | September 2, 2016 | The initial version |
| 2.0 | November 18, 2016 | 1. Add a new treatment arms in the treating component: NCT02959619 (X-396/Ensartinib);  2. Expand the age range of eligible patients. |
| 2.1 | Jan 12, 2017 | 1. Adjust the actionability level of RET fusion from level 3 to level 2 based on latest evidence regarding cabozantinib and the OncoKB recommendation. |
| 3.0 | June 7, 2017 | 1. Add a new treatment arms in the treating component: CTR20170407 (ACC006). |
| 3.1 | June 28, 2018 | 1. Adjust the actionability level of NTRK fusion from level 3 to level 2 based on latest evidence regarding larotrectinib and the OncoKB recommendation. |
| 4.0 | June 28, 2018 | 1. Extend the recruitment period of the study from December 31, 2019 to December 31, 2020, in order to reach the planned sample size. |
| 5.0 | October 22, 2018 | 1. Add a new treatment arms in the treating component: CTR20180977 (ML007);  2. Adjust the actionability level of ERBB2 mutation from level 3 to level 2 based on latest evidence regarding T-DM1 and pyrotinib and the OncoKB recommendation. |
| 5.1 | Jan 24, 2018 | 1. Adjust the actionability level of ROS1 fusion from level 2 to level 1 based on the NMPA approval of crizotinib for this indication in China. |
| 5.2 | July 10, 2019 | 1. Adjust the actionability level of KRAS G12C mutation from level 4 to level 3 based on latest evidence regarding AMG510 and the OncoKB recommendation. |

**TABLE OF CONTENTS**

[**Protocol Amendments Records** 4](#_Toc64191862)

[**1.** **STUDY SYNOPSIS** 8](#_Toc64191863)

[**2.** **BACKGROUND AND RATIONALE** 10](#_Toc64191864)

[**2.1 Non-small cell lung cancer** 10](#_Toc64191865)

[**2.2 Molecular targeted therapy in NSCLC** 11](#_Toc64191866)

[**2.3** **The role of comprehensive genomic profiling** 12](#_Toc64191867)

[**3.** **STUDY OBJECTIVES** 13](#_Toc64191868)

[**3.1 Primary Objectives** 13](#_Toc64191869)

[**3.2 Secondary Objective** 13](#_Toc64191870)

[**4.** **STUDY POPULATION** 14](#_Toc64191871)

[**4.1 Screening Component** 14](#_Toc64191872)

[**4.1.1 Inclusion Criteria** 14](#_Toc64191873)

[**4.1.2 Exclusion Criteria** 15](#_Toc64191874)

[**4.2 Treating Component** 16](#_Toc64191875)

[**4.2.1 Inclusion Criteria** 16](#_Toc64191876)

[**4.2.2 Exclusion Criteria** 16](#_Toc64191877)

[**4.2.3 Withdrawal Criteria** 17](#_Toc64191878)

[**4.2.4 Discontinuity or Termination Criteria** 17](#_Toc64191879)

[**5.** **OUTCOME MEASURES** 18](#_Toc64191880)

[**5.1 Impacts of comprehensive genomic profiling on treatment selection** 18](#_Toc64191881)

[**5.2 Impacts of comprehensive genomic profiling on clinical trial enrollment** 18](#_Toc64191882)

[**5.3** **Progression-free survival time (PFS)** 18](#_Toc64191883)

[**5.4 Overall survival time (OS)** 18](#_Toc64191884)

[**5.5 Outcomes of associated clinical trials** 19](#_Toc64191885)

[**6.** **STATSTICAL CONSIDERATIONS** 19](#_Toc64191886)

[**6.1 Planned sample size** 19](#_Toc64191887)

[**6.2 Endpoint analysis** 19](#_Toc64191888)

[**6.3 Radiological examination plan** 19](#_Toc64191889)

[**7.** **STUDY PROCEDURE** 20](#_Toc64191890)

[**7.1 Screening** 20](#_Toc64191891)

[**7.2 Patient consent** 20](#_Toc64191892)

[**7.3 Genomic profiling and results reporting** 20](#_Toc64191893)

[**7.4 Treatment allocation** 21](#_Toc64191894)

[**7.5 Treatment and follow-up schema** 22](#_Toc64191895)

[**9.** **REFERENCE LIST** 23](#_Toc64191896)

[**APPENDIX I. ECOG PERFORMANCE STATUS** 25](#_Toc64191897)

[**APPENDIX II. RECIST VERSION 1.1** 26](#_Toc64191898)

1. **STUDY SYNOPSIS**

**Design**

This is a nonrandomized study contained a screening component and a treating component. The screening component enrolled patients with advanced non-small cell lung cancer (NSCLC) and used hybridization capture-based next-generation sequencing panels to identify potentially actionable alterations. The treating component contained nine independently designed and registered clinical trials. Patients carrying potentially actionable alterations were allocated to associated trials based on the biomarker-enrichment strategy of each trial. Patients with actionable alterations but were ineligible or refused to participate in associated trials were treated at the discretion of their treating physicians and followed up.

**Objectives**

**Primary Objectives**

- To determine whether personalized therapy based on comprehensive genomic profiling is feasible for Chinese patients with advanced NSCLC.
- To determine the role of comprehensive genomic profiling in treatment selection and clinical trial enrollment.
- To evaluate the clinical impact of comprehensive genomic profiling-based personalized therapy in Chinese patients with advanced NSCLC.

**Secondary Objectives**

- To evaluate the safety and efficacy of the investigated agents in associated clinical trials in patients with advanced NSCLC.

**Outcomes**

- Impacts of comprehensive genomic profiling on treatment selection
- Impacts of comprehensive genomic profiling on clinical trial enrollment
- Progression-free survival time (PFS)
- Overall survival time (OS)
- Outcomes of associated clinical trials

**Study Population**

**Key inclusion criteria**

1. Age≥18 years at the time of consent;
2. Histological or cytologically confirmed, stage III (not suitable for radical radiotherapy or surgery) or stage IV NSCLC;
3. Eastern Cooperative Group ECOG) Performance Status score of 0-2;
4. Could provide tumor specimen adequate for comprehensive genomic profiling (detailed in Section 4.1);
5. Computed Tomography (CT) or Magnetic Resonance Imaging (MRI) scan of head, chest and abdomen within 28 days of treatment demonstrating measurable disease as per Response Evaluation Criteria In Solid Tumors (RECIST) version 1.1;
6. Adequate organ function;
7. Male patients willing to use adequate contraceptive measures; female patients who are not of child-bearing potential, and female patients of child-bearing potential who agree to use adequate contraceptive measures;
8. A life expectancy of at least 3 months;
9. Provided written informed consent;
10. Patients enrolled into associated arms should also meet the specific inclusion criteria of the corresponding trial.

**Key exclusion criteria**

1. Previous or concurrent malignancies diagnosed within the past 5 years, except for adequately treated basal cell carcinoma of the skin and in situ carcinoma of the uterine cervix;
2. Any major surgery or radiotherapy within 4 weeks prior to enrollment;
3. Prior stem cell transplant;
4. Severe or uncontrolled disease in respiratory or cardiovascular system;
5. Uncontrollable symptoms of brain metastases, spinal cord compression, carcinomatous meningitis, or brain or leptomeningeal disease detected by CT or MRI during the past 8 weeks;
6. Unwilling or unbale to comply with the study protocol and follow-up procedures;
7. Concurrent condition evaluated by investigator would jeopardize compliance with the protocol or would impart excessive risk associated with study participation that would make it inappropriate for the patient to be enrolled.

**Planned Sample Size**

2000 patients

**Study Period**

Recruitment: October 1, 2016 to December 31, 2020

Study: October 1, 2016 to December 31, 2023

1. **BACKGROUND AND RATIONALE**

## **2.1 Non-small cell lung cancer**

Lung cancer is the leading cause of cancer-related deaths in China and worldwide. Non small-cell lung cancer (NSCLC) accounts for 80-85% of lung cancer cases. About 30-40% of newly diagnosed cases were advanced diseases. About 69% of advanced lung adenocarcinoma patients harbor a potentially actionable alteration. Figure 1 (Hirsch 2017) presents the frequency of molecular alterations in driver oncogenes in lung adenocarcinoma. Epidermal growth factor receptor (EGFR) is one of the most classic oncogenes in NSCLC. EGFR alterations occur in 40% of Asian patients and 10-20% of non-east Asian patients. The majority of patients carried EGFR exon 19 deletions or L858R mutations, both are sensitive to EGFR tyrosine kinase inhibitors (TKI). Approximately 10-12% of EGFR-mutant NSCLC harbor an exon 20 in-frame insertion, which are resistant to currently approved EGFR TKIs. Anaplastic lymphoma kinase (ALK) fusion is observed in 2-7% of NSCLC. Most of these patients are Asian, never- or light smokers and men. ROS1 fusion, MET alterations, BRAF mutations and RET fusions are respectively observed in 1-2%, 3%, 3-5% and 2% of NSCLC, and are mutually exclusive with each other. HER2 mutations occur in 2-4% of NSCLC, most of which are exon 20 in-frame insertions. KRAS mutations are oncogenic alterations frequently found in non-Asian patients. It was deemed an undruggable target until the recent report of a KRAS G12C inhibitor.


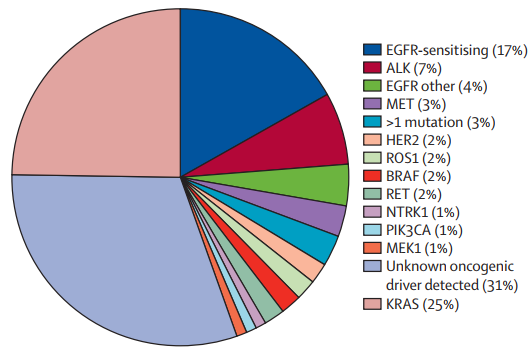


Figure 1. Distribution of potentially actionable alterations in oncogenic drivers in lung adenocarcinoma (Adapted from Hirsch 2017).

**2.2 Molecular targeted therapy in NSCLC**

Advances in tumor genomics through next-generation sequencing and the emergence of molecularly targeted agents have significantly transformed the treatment landscape of advanced NSCLC. EGFR TKIs have been established as the standard first-line treatment in EGFR-mutant NSCLC and significantly extended progression-free survival and overall survival in comparison to chemotherapy. For patients with de-novo or acquired resistance to the first- and second-generation EGFR TKIs, molecular analysis of the resistant specimen unveiled the mechanism of resistance and led to the development of third-generation EGFR TKIs and combination strategies. Meanwhile, several ALK TKIs, including crizotinib, ceritinib, and alectinib, were developed and constitute the backbone of treatment for advanced, ALK-positive NSCLC. Crizotinib was also approved as the standard targeted therapy in previously untreated, advanced NSCLC patients carrying ROS1 fusions. Recently, molecularly targeted agents with high efficacy and acceptable tolerability have been developed and approved by the FDA targeting advanced NSCLC carrying MET alterations, RET fusions, BRAF mutations and NTRK fusions, respectively. Some of these agents are being investigated in clinical trials in China and might be approved by the NMPA (National Medical Products Administration) in the near future. In terms of HER2 mutations and EGFR exon 20 insertions, there are several targeted agents, including small molecular TKIs, antibody-drug conjugates and bispecific antibodies being actively investigated in clinical trials. And some of them have reported promising results.

- 1. **The role of comprehensive genomic profiling**

Precision cancer medicine has become a trending concept in clinical oncology recently. The key hypothesis behind this practice is that comprehensive molecular analysis of tumor specimen from individual patients will direct treatment selection, effectively curb tumor growth, and eventually improve clinical outcomes. Given the success achieved by targeted therapies in several molecularly-defined subgroups of NSCLC, precision cancer medicine based on large-panel next-generation sequencing has been advocated for advanced NSCLC by the National Comprehensive Cancer Network (NCCN) and International Association for the Study of Lung Cancer (IASLC). However, the clinical impact of comprehensive genomic profiling for patients with advanced NSCLC remains controversial. The SHIVA study, a randomized trial comparing genomic profiling-based targeted therapy versus conventional therapy reported a negative progression-free survival result. A retrospective study also observed a lack of significant association between board-based genomic sequencing and survival extension. While studies conducted by the Lung Cancer Mutational Consortium and MD Anderson Cancer Center Initiative reported survival benefits in patients receiving genomic profiling-based matched therapies. Meanwhile, despite the extensive data, Asian patients were underrepresented in existed studies. Experiences with personalized therapy in developing countries is also lacking. Therefore, a better understanding of the feasibility and clinical impacts of comprehensive genomic profiling-based personalized therapy is Chinese patients with advanced NSCLC is needed.

1. **STUDY OBJECTIVES**

## **3.1 Primary Objectives**

- To determine the feasibility of personalized therapy based on comprehensive genomic profiling for Chinese patients with advanced NSCLC.
- To determine the role of comprehensive genomic profiling in treatment selection and clinical trial enrollment.
- To evaluate the clinical impact of comprehensive genomic profiling-based personalized therapy in Chinese patients with advanced NSCLC in terms of progression-free survival and overall survival.

## **3.2 Secondary Objective**

- **Trial 1:** To evaluate the safety and efficacy of Apatinib in combination with Gefitinib as compared to placebo in combination with Gefitinib in participants with stage IIIB-IV non-squamous NSCLC harboring an activating EGFR mutation (Del19 and L858R).
- **Trial 2:** To evaluate the safety and efficacy of CT053PTSA (Ningetinib) in combination with gefitinib in patients with EGFR mutation, T790M negative NSCLC who have progressed after EGFR TKI treatment.
- **Trial 3:** To assess the safety, tolerability, pharmacokinetics and anti-tumor activity measured by overall response rate of AC0010 Maleate Capsule (Avitinib) in local advanced or metastatic non-small cell lung cancer patients with EGFR mutation or T790M drug-resistant mutation.
- **Trial 4:** To evaluate the safety, tolerability, single-dose and successive-dose pharmacokinetics and anti-tumor activity measured by ORR, DCR and PFS of ML-007 in patients with advanced solid tumors.
- **Trial 5:** To determine the largest amount of ensartinib that can be safely given to humans. An expansion phase will be conducted to assess the preliminary anti-tumor activity in ALK-positive non-small cell lung cancer once the recommended Phase 2 dose has been established.
- **Trial 6:** To evaluate the safety, tolerability, single-dose and successive-dose pharmacokinetics and anti-tumor activity measured by ORR, DCR and PFS of HMPL-504 (Volitinib) in patients with advanced solid tumors.
- **Trial 7:** To characterize the safety, tolerability and pharmacokinetics of single-dose and successive-dose ACC006 in subjects with solid tumor; To determine MTD and DLT.

1. **STUDY POPULATION**

## **4.1 Screening Component**

### **4.1.1 Inclusion Criteria**

Patients who meet all of the following items will be eligible for enrollment:

1. Age≥18 years at the time of consent;
2. Histological or cytologically confirmed, stage III (not suitable for radical radiotherapy or surgery) or stage IV NSCLC;
3. Eastern Cooperative Group ECOG) Performance Status score of 0-2;
4. Could provide tumor specimen adequate for comprehensive genomic profiling. Definition of sample adequate to submit for testing:

- Biopsy or paraffin-embedded cytology cell block specimen with surplus tissue available for genomic profiling using hybridization capture-based next-generation sequencing panels;
- Samples should contain at least 20% of tumor tissue;
- Origin from either the primary tumor or a site of metastasis. Samples derived from bone metastasis will not be accepted;
- Matching blood sample is not mandatory.

Condition that a re-biopsy is mandatory:

- Patients with recurrent diseases that relapsed more than 6 months after surgery;
- Patients who had progressive diseases after a targeted therapy in the context of the PREVAIL program.

1. Computed Tomography (CT) or Magnetic Resonance Imaging (MRI) scan of head, chest and abdomen within 28 days of treatment demonstrating measurable disease as per Response Evaluation Criteria in Solid Tumors (RECIST) version 1.1;
2. Adequate organ function:

- Blood routine: HB ≥ 90 g/L (no blood transfusion within 14 days); ANC ≥ 1.5 x 10 9 / L; PLT ≥ 100 x 109 /L
- Blood chemistry: BIL, ALT and AST, serum Cr within normal limits or creatinine clearance ≥ 60 mL/min (calculated according to Cockcroft-Gault formula)
- Male patients: CLcr (glomerular filtration rate) = [(140-age (years) × weight (kg))/[72 × serum creatinine (mg/dL)];
- Female patients: CLcr (glomerular filtration rate) = 0.85 × CLcr in male patients;
- Triglycerides ≤ 3.42 mmol/L, cholesterol ≤ 7.75 mmol/L;
- Doppler ultrasound: left ventricular ejection fraction (LVEF) ≥ 50%.

1. Male patients willing to use adequate contraceptive measures; female patients who are not of child-bearing potential, and female patients of child-bearing potential who agree to use adequate contraceptive measures;
2. A life expectancy of at least 3 months;
3. Provided written informed consent;

### **4.1.2 Exclusion Criteria**

Patients will not be eligible for enrollment in this study for any of the following:

1. Previous or concurrent malignancies diagnosed within the past 5 years, except for adequately treated basal cell carcinoma of the skin and in situ carcinoma of the uterine cervix;
2. Any major surgery or radiotherapy within 4 weeks prior to enrollment;
3. Prior stem cell transplant;
4. Severe or uncontrolled disease in respiratory or cardiovascular system, including:

- Uncontrolled chronic obstructive pulmonary disease, history of acute exacerbation within the past 6 months;
- Uncontrolled asthma that required the administration of steroids;
- Uncontrolled hypertension: systolic blood pressure ≥ 140 mmHg, diastolic blood pressure ≥ 90 mmHg under one drug therapy;
- Myocardial ischemia or myocardial infarction;
- Arrhythmia, including QT interval ≥ 470ms;
- Grade I cardiac insufficiency;

1. Uncontrollable symptoms of brain metastases, spinal cord compression, carcinomatous meningitis, or brain or leptomeningeal disease detected by CT or MRI during the past 8 weeks.
2. Unwilling or unbale to comply with the study protocol and follow-up procedures;
3. Any condition evaluated by investigator would jeopardize compliance with the protocol or would impart excessive risk associated with study participation that would make it inappropriate for the patient to be enrolled.

## **4.2 Treating Component**

### **4.2.1 Inclusion Criteria**

Patients who meet all of the following items will be eligible for enrollment:

1. Patients who met the inclusion criteria listed in Section 4.1.1 and were enrolled into the screening component;
2. Complete comprehensive genomic profiling of tumor;
3. Genomic profiling of the tumor reported potentially actionable alterations based on the OncoKB dataset (<https://www.oncokb.org/>);
4. For patients who were assigned to specific associated trials, should meet all the inclusion criteria of the corresponding trial;
5. The patient voluntarily joined the associated trial, signed the informed consent form, and had good compliance.

### **4.2.2 Exclusion Criteria**

Patients will not be eligible for enrollment in this study for any of the following:

1. Failed to complete comprehensive genomic profiling of tumor due to any reason;
2. Genomic profiling of the tumor detected no potentially actionable alterations based on the OncoKB dataset (<https://www.oncokb.org/>);
3. Any factors that may affect drug administration and absorption, such as inability to swallow, received gastrointestinal resection, chronic diarrhea, and intestinal obstruction, etc.;
4. Patients who were assigned to specific associated trials but meet any of the exclusion criteria of the corresponding trial;
5. For patients who treated outside the associated clinical trials, unwilling or unable to comply with the follow-up procedures;
6. Any condition evaluated by investigator would jeopardize compliance with the protocol or would impart excessive risk associated with study participation that would make it inappropriate for the patient to be enrolled.

### **4.2.3 Withdrawal Criteria**

1. Poor patient compliance;
2. Using of other drugs that affect the judgment of tolerability and pharmacokinetic analysis according the investigator;
3. Serious adverse events or unintended pregnancy;
4. Patients who are unwilling to continue the clinical trial and voluntarily request withdrawal;
5. The investigator considered it necessary to discontinue the study.

### **4.2.4 Discontinuity or Termination Criteria**

1. Disease progression indicated by radiological examinations or symptom exacerbation;
2. Presence of dose limiting toxicity (DLT);
3. **OUTCOME MEASURES**

## **5.1 Impacts of comprehensive genomic profiling on treatment selection**

The proportion of patients treated with genomic profiling-matched therapy/The proportion of patients who completed genomic profiling of the tumor and received genomic profiling results.

## **5.2 Impacts of comprehensive genomic profiling on clinical trial enrollment**

The proportion of patients enrolled into the associated clinical trials testing a matched targeted therapy/The proportion of patients who completed genomic profiling of the tumor and received genomic profiling results.

- 1. **Progression-free survival time (PFS)**

Progression-free survival is defined as the time from the initiation of treatment to the date of CT or MRI scan that first recorded progressive disease or date of death without previously recorded progression. For patients who are enrolled into associated clinical trials, they receive CT or MRI scans according to the schedule of the corresponding protocol. For patients who harbor potentially actionable alterations but are treated outside the associated trials, CT or MRI scans are performed every 6 weeks from baseline until disease progression or unacceptable toxicity. Patients who are alive with no recorded progression at the time of analysis will be censored at the date of their last CT or MRI scan.

## **5.4 Overall survival time (OS)**

Overall survival is defined as the time from the initiation of treatment to the date of death for any cause. Patients who are alive at the time of analysis will be censored at the date of last follow-up.

## **5.5 Outcomes of associated clinical trials**

Outcomes specified in the protocols of associated clinical trials (Arm 1-9).

1. **STATSTICAL CONSIDERATIONS**

## **6.1 Planned sample size**

Planned sample size: 2000 patients

Rationale: The planned recruitment period for the study is from October 1, 2016 to December 31, 2020. Considering the number of patients presented to the clinic at Sun Yat-Sen University Cancer Center and the number of patients diagnosed with locally advanced or metastatic NSCLC, we considered that 2000 is a rational sample size.

## **6.2 Endpoint analysis**

Feasibility outcomes as defined in Section 5.1-5.2 will be based on the eligible and treated patients. Survival outcomes as defined in Section 5.3-5.4 will be estimated using the Kaplan-Meier method. Population for survival outcome analysis are those who have potentially actionable alterations identified and enrolled into associated treatment arms, or treated off trial but are willing to comply with follow-up procedures.

## **6.3 Radiological examination plan**

Tumor responses will be evaluated using CT or MRI scans, which will be collected according to arm-specific protocol or every 6 weeks for those treated off trial. These images will be archived centrally and analyzed arm-specifically. When adequate radiological, genomic data are available, cross-arm analysis will be performed.

1. **STUDY PROCEDURE**

**7.1 Screening**

Potentially eligible patients are those who present to the clinic with a diagnosis of locally advanced (inappropriate for definitive surgery or radiotherapy) or metastatic NSCLC, have a ECOG performance score of 0-2 and no serious co-morbidities. These patients will be further assessed for eligibility and be asked for consent to participate in the study.

**7.2 Patient consent**

A written informed consent for providing tumor samples and genomic sequencing will be obtained from each participant. If a re-biopsy is needed, a consent for re-biopsy will be obtained. If the patient has a potentially actionable genetic alteration based on the OncoKB dataset and is eligible for associated treatment arms, an arm-specific inform consent will be signed. If the patient has a potentially actionable genetic alteration but is ineligible or refuse to participate in associated treatment arms,

**7.3 Genomic profiling and results reporting**

Patients who meet the inclusion criteria of the screening component and give consent will provide their tumor samples, either from their diagnostic tumor specimen extraction or a re-biopsy. The sample will then be sent to one of the two designated genetic testing laboratories for nucleic acid extraction and genomic profiling using hybridization capture-based next-generation sequencing panels. Characterized and reported genomic alterations include SNVs, CNVs, indels and translocations. Surplus samples will be destructed after nucleic acid extraction. Once the genomic profiling is completed, the result will be made available to the patient and the treating physician. A hardcopy of the genomic profiling result will also be sent to the patient. Profiling results will also be stored at the two laboratories. The study is planned to end recruitment on December 31, 2020, when all profiling results will be submitted and stored at China National Center for Bioinformation for future ethically-approved research use.

**7.4 Treatment allocation**

Patients carrying potentially actionable genomic alterations will be allocated to associated clinical trials testing a matched targeted therapy. If more than one actionable alteration is identified, the decision will be made according to the alteration with the highest actionability level (detailed below). Patients carrying potentially actionable alterations who are ineligible or unwilling to participate in associated trials, but are willing to comply with the follow-up procedures will be treated with matched targeted therapy or non-matched therapy at the discretion of the treating physician and stay in the study for clinical outcome analysis. Actionability of genomic alterations and the level of evidence were determined based on the OncoKB dataset and drug approval status in mainland China:

- Level 1 (Genomic alterations with approved targeted therapies for this indication in mainland China):

EGFR 19del/L858R, EGFR 19del/L858R+T790M, EGFR G719X/L861Q/S768I, ALK fusion;

- Level 2 (Genomic alterations with targeted agents as investigational new drug for this indication in mainland China):

ROS1 fusion, BRAF V600E mutations, MET exon 14 deletion/skipping mutations/amplifications;

- Level 3 (Genomic alterations with potential targeted agents supported by clinical evidence):

ARAF mutations, EGFR exon 20 insertions/exon 19 insertions/kinase domain duplications, ERBB2 mutations/exon 20 insertions, RET fusion, NTRK 1/2/3 fusions, MAP2K1 mutations, EGFR L718V/D761Y/C797S, KIT mutations

- Level 4 (Genomic alterations with potential drug supported by clinical evidence in another indication):

BRAF non-V600E mutations, CDK4 amplifications, CDKN2A mutations, FGFR1/2/3 translocations/mutations, NF1 mutations, KRAS mutations, BRCA, CDK12, PI3K pathway mutations (PIK3CA mutations, AKT mutations/amplifications, MTOR mutations, PTEN loss/mutations/deletions, TSC1/2 mutations), NF1 mutations.

**7.5 Treatment and follow-up schema**

Patients being enrolled into associated treatment arms will be treating according to the treatment schema detailed in the arm-specific protocols. Treatment compliance will be monitored by patient diary cards every cycle. For patients who are enrolled into associated treatment arms, dose modification will be conducted according to the instructions detailed in the arm-specific protocols. For patients who are treated off trial but continue to stay in the study for follow-up, dose modification will be conducted based on clinical routines. Patients who have potentially actionable alterations identified and received targeted therapies, either on associated treatment arms or off trial, will be followed for survival for 3 years.

1. **ETHICS**

The study will be conducted in accordance with the Declaration of Helsinki and relevant Chinese clinical trial study norms and regulations. The study protocol is formulated before the start of the clinical trial, and submitted to the Ethics Committee of Sun Yat-Sen University Cancer Center for review and approval. During the actual implementation of the clinical trial, if amendments are required to this protocol, the revised protocol will be submitted to the Ethics Committee for approval before implementation. Before the start of the study, the investigator must provide the patient with detailed information about the study, including the nature of the study, the purpose, possible benefits and risks, other available treatment options and the rights and obligations of the patient. The patient will be considered as a participant until he/she fully understands and signs the Informed Consent Form. Genomic profiling results will be made available to the patient regardless whether he/she decide to stay in the study population for follow-up.

1. **REFERENCE LIST**
2. Hirsch FR, Scagliotti GV, Mulshine JL, et al. Lung cancer: current therapies and new targeted treatments. The Lancet. 2017;389(10066):299-311.
3. Robichaux JP, Elamin YY, Tan Z, et al. Mechanisms and clinical activity of an EGFR and HER2 exon 20-selective kinase inhibitor in non-small cell lung cancer. Nat Med. 2018;24(5):638-646.
4. Tannock IF, Hickman JA. Limits to Personalized Cancer Medicine. The New England journal of medicine. 2016;357:1289-1294.
5. Ettinger DS, Wood DE, Aggarwal C, et al. NCCN Guidelines Insights: Non-Small Cell Lung Cancer, Version 1.2020. J Natl Compr Canc Netw. 2019;17(12):1464-1472.
6. Lindeman NI, Cagle PT, Aisner DL, et al. Updated Molecular Testing Guideline for the Selection of Lung Cancer Patients for Treatment With Targeted Tyrosine Kinase Inhibitors: Guideline From the College of American Pathologists, the International Association for the Study of Lung Cancer, and the Association for Molecular Pathology. Journal of thoracic oncology: official publication of the International Association for the Study of Lung Cancer. 2018;13(3):323-358.
7. Le Tourneau C, Delord J-P, Gonçalves A, et al. Molecularly targeted therapy based on tumor molecular profiling versus conventional therapy for advanced cancer (SHIVA): a multicentre, open-label, proof-of-concept, randomised, controlled phase 2 trial. The Lancet Oncology. 2015;16(13):1324-1334.
8. Presley CJ, Tang D, Soulos PR, et al. Association of Broad-Based Genomic Sequencing With Survival Among Patients With Advanced Non–Small Cell Lung Cancer in the Community Oncology Setting. Jama. 2018;320(5).
9. Tsimberidou AM, Iskander NG, Hong DS, et al. Personalized medicine in a phase I clinical trials program: the MD Anderson Cancer Center initiative. Clinical cancer research: an official journal of the American Association for Cancer Research. 2012;18(22):6373-6383.
10. Kris MG, Johnson BE, Berry LD, et al. Using multiplexed assays of oncogenic drivers in lung cancers to select targeted drugs. Jama. 2014;311(19):1998-2006.
11. Eisenhauer EA, Therasse P, Bogaerts J, et al. New response evaluation criteria in solid tumors: revised RECIST guideline (version 1.1). European journal of cancer (Oxford, England: 1990). 2009;45(2):228-247.
12. Chakravarty D, Gao J, Phillips S, et al. OncoKB: A Precision Oncology Knowledge Base. 2017(1):1-16.

# **APPENDIX I.**

# **ECOG PERFORMANCE STATUS***

| **Grade** | **ECOG Performance status** |
| --- | --- |
| 0 | Fully active, able to carry on all pre-disease performance without restriction |
| 1 | Restricted in physically strenuous activity but ambulatory and able to carry out work of a light or sedentary nature, e.g., light house work, office work |
| 2 | Ambulatory and capable of all selfcare but unable to carry out any work activities. Up and about more than 50% of waking hours |
| 3 | Capable of only limited selfcare, confined to bed or chair more than 50% of waking hours |
| 4 | Completely disabled. Cannot carry on any selfcare. Totally confined to bed or chair |
| 5 | Dead |

*As published in Oken MM, Creech RH, Tormey DC, et al. Toxicity and response criteria of the Eastern Cooperative Oncology Group. American journal of clinical oncology. 1982;5(6):649-655.

# **APPENDIX II.**

# **RESPONSE EVALUATION CRITERIA IN SOLID TUMORS (RECIST) VERSION 1.1**

Response and progression will be evaluated in this study using the Response Evaluation Criteria in Solid Tumors (RECIST) guideline (version 1.1) as published in Eisenhauer EA, Therasse P, Bogaerts J, et al. New response evaluation criteria in solid tumors: revised RECIST guideline (version 1.1). European journal of cancer (Oxford, England: 1990). 2009;45(2):228-247.

The following contains excerpts from the RECIST version 1.1 and specific requirements in this study. A complete copy of the RECIST version 1.1 guideline is available at: <http://www.eortc.be/recist/RECIST_EORTC_NCI_AACR_OCtober_2008.pdf>.

**Measurability of tumor at baseline**

Measurable: Tumor lesions should be accurately measured in at least one dimension (longest diameter in the plane of measurement is to be recorded) with a minimum size of 10 mm by CT scan. Malignant lymph nodes should be ≥15 mm in short axis when assessed by CT scan. CT scan used for evaluation should have slice thickness no greater than 5mm.

Non-measurable: All other lesions, including small lesions (longest diameter <10 mm or pathological lymph nodes with ≥10 to <15 mm short axis) as well as truly non-measurable lesions. Lesions considered truly non-measurable include: leptomeningeal disease, ascites, pleural or pericardial effusion, inflammatory breast disease, lymphangitic involvement of skin or lung, abdominal masses/abdominal organomegaly identified by physical exam that is not measurable by reproducible imaging techniques.

Special considerations regarding lesion measurability:

Bone lesions: Bone scan, PET scan or plain films are not considered adequate imaging techniques to measure bone lesions. However, these techniques can be used to confirm the presence or disappearance of bone lesions. Bone scan, PET scan or plain films are not considered adequate imaging techniques to measure bone lesions. However, these techniques can be used to confirm the presence or disappearance of bone lesions. Blastic bone lesions are non-measurable.

Cystic lesions: Lesions that meet the criteria for radiographically defined simple cysts should not be considered as malignant lesions (neither measurable nor non-measurable) since they are, by definition, simple cysts. ‘Cystic lesions’ thought to represent cystic metastases can be considered as measurable lesions, if they meet the definition of measurability described above. However, if noncystic lesions are present in the same patient, these are preferred for selection as target lesions.

Lesions with prior local treatment: Tumor lesions situated in a previously irradiated area, or in an area subjected to other loco-regional therapy, are usually not considered measurable unless there has been demonstrated progression in the lesion. Study protocols should detail the conditions under which such lesions would be considered measurable.

**Methods of measurement**

Measurement of lesions: All measurements should be recorded in metric notation, using calipers if clinically assessed. All baseline evaluations should be performed as close as possible to the treatment start and never more than 4 weeks before the beginning of the treatment.

Method of assessment: Chest X-ray is not acceptable for tumor assessment in this study. All patients are required to perform a chest + upper abdomen CT scan at baseline. CT scans should have scan thickness no greater than 5mm. MRI is required for patients with central nervous system metastasis. PET-CT is acceptable for baseline assessment but CT scans are required for follow-up evaluations.

**Tumor response evaluation**

Baseline documentation of ‘target’ and ‘non-target’ lesions: When more than one measurable lesion is present at baseline all lesions up to a maximum of five lesions total (and a maximum of two lesions per organ) representative of all involved organs should be identified as target lesions and will be recorded and measured at baseline. Target lesions should be selected on the basis of their size (lesions with the longest diameter), be representative of all involved organs, but in addition should be those that lend themselves to reproducible repeated measurements.

A sum of the diameters (longest for non-nodal lesions, short axis for nodal lesions) for all target lesions will be calculated and reported as the baseline sum diameters.

All other lesions (or sites of disease) including pathological lymph nodes should be identified as non-target lesions and should also be recorded at baseline.

Response criteria:

Evaluation of target lesions:

Complete Response (CR): Disappearance of all target lesions. Any pathological lymph nodes (whether target or non-target) must have reduction in short axis to <10 mm.

Partial Response (PR): At least a 30% decrease in the sum of diameters of target lesions, taking as reference the baseline sum diameters.

Progressive Disease (PD): At least a 20% increase in the sum of diameters of target lesions, taking as reference the smallest sum on study (this includes the baseline sum if that is the smallest on study). In addition to the relative increase of 20%, the sum must also demonstrate an absolute increase of at least 5 mm. The appearance of one or more new lesions is also considered progression.

Stable Disease (SD): Neither sufficient shrinkage to qualify for PR nor sufficient increase to qualify for PD, taking as reference the smallest sum diameters while on study.

Evaluation of non-target lesions:

Complete Response (CR): Disappearance of all non-target lesions and normalization of tumour marker level. All lymph nodes must be non-pathological in size (<10 mm short axis).

Non-CR/Non-PD: Persistence of one or more non-target lesion(s) and/or maintenance of tumour marker level above the normal limits.

Progressive Disease (PD): Unequivocal progression (see comments below) of existing non-target lesions. The appearance of one or more new lesions is also considered progression.

Evaluation of best overall response:

| **Target Lesions** | **Non-Target Lesions** | **New Lesions** | **Overall Response** |
| --- | --- | --- | --- |
| CR | CR | No | CR |
| CR | Non CR/Non-PD | No | PR |
| CR | Not evaluated | No | PR |
| PR | Non CR/Non PD/not evaluated | No | PR |
| SD | Non CR/Non PD/not evaluated | No | SD |
| PD | Any | Yes or No | PD |
| Any | PD | Yes or No | PD |
| Any | Any | Yes | PD |
